# Revision of tibial TKA components: bone loss is independent of cementing type and technique: an in vitro cadaver study

**DOI:** 10.1186/1471-2474-12-6

**Published:** 2011-01-10

**Authors:** Turgay Efe, Jens Figiel, David Sibbert, Susanne Fuchs-Winkelmann, Carsten O Tibesku, Nina Timmesfeld, Jürgen RJR Paletta, Adrian Skwara

**Affiliations:** 1 Department of Orthopaedics and Rheumatology, University Hospital Marburg, Baldingerstrasse, 35043 Marburg, Germany; 2 Department of Diagnostic Radiology, University Hospital Marburg, Baldingerstrasse, 35043 Marburg, Germany; 3 sporthopaedicum Straubing, Bahnhofplatz 8, 94315 Straubing, Germany; 4 Institute of Medical Biometry and Epidemiology, Philipps-University Marburg, Bunsenstrasse 3, 35037 Marburg, Germany

## Abstract

**Background:**

Different bone cements and various cementation techniques can lead to different bone loss in revision surgery. We investigated the degree of tibial bone loss depending on different cements and techniques.

**Methods:**

30 tibia specimens were matched into three groups (10 each). In all cases Genesis II tibia component were implanted. In two groups, the tibia base plate alone was cemented with Palacos^® ^R+G and Refobacin^® ^Bone Cement R. In the third group, both tibial base plate and tibial stem were cemented with Palacos^® ^R+G. Afterwards, the specimens were axial loaded with 2000 N for 10,000 cycles. Tibial components were explanted and the required time to explantation was recorded. Bone loss after explantation was measured by CT.

**Results:**

On CT, there was no significant difference in bone loss between cementing techniques (p = 0.077; 95% CI -1.14 - 21.03) or the cements themselves (p = 0.345; 95% CI -6.05 - 16.70). The required time to explantation was 170.6 ± 54.89, 228.7 ± 84.5, and 145.7 ± 73.0 seconds in the first, second, and third groups, respectively.

**Conclusions:**

Cement technique and type do not influence tibial bone loss in simulated revision surgery of the tibial component in knee arthroplasty.

## Background

Primary cemented total knee arthroplasty (TKA) is a well-established procedure with excellent clinical results [1,2]. Enhanced indications for joint arthroplasty have come about as a result of patients' desires for self-sufficiency and improved quality of life. However, even as the number of revisions are unchanged or even decreasing, the increasing number of TKAs will result in more revision procedures in the future.

Revision total knee arthroplasty presents a clinical challenge. A significant problem is cement removal and handling in the face of bone deficiency. To provide stable implant fixation and to reestablish the correct joint line bony defects can be treated with cement, modular augments, custom-made implants, and bone grafts [3-5]. Bone cements of different manufacturers have the same functional principles but have differences in chemical composition [6] which can effect their material properties. The varying viscosity of bone cements can lead to different depths of intrusion. By using horizontal and full cement techniques, different areas of the bone are covered. This can lead to different degrees of bone loss during revision surgery. Because of these complications, and the lack of scientific verification, we investigated whether different bone cements and cement techniques affect tibial bone loss.

## Methods

This project was performed in accordance to the Helsinki Declaration, and to local legislation. An ethical approval was not necessary. The Experiments were conducted on 30 fresh-frozen tibiae from donors with an average age of 79.6 ± 9.4 years. They were separated into three groups of 10 specimens each, and matched according to gender, age, and tibia dimension. Prior to implantation, plain radiographs were taken to exclude specimens with abnormalities and osseous lesions; none were evident. Each tibia was assigned to a "treatment group" using random permutated blocks. In the first group, 10 tibial trays were cemented horizontally using Palacos^® ^R+G (Heraeus, Wehrheim, Germany). In the second group, 10 tibial components were cemented horizontally using Refobacin^® ^Bone Cement R (Biomet, Warsaw, USA). In the third group, 10 tibial components were fully cemented using Palacos^® ^R+G (Heraeus, Wehrheim, Germany). This technique includes a cement application under the tibial base plate and around the tibial stem.

The original cemented tibial tray design of the Genesis II (Smith & Nephew, Schenefeld, Germany) TKA system was used. Tibial specimens were prepared according to the manufacturer's guidelines with the original Genesis II instruments for cemented implants. Prior to osseous preparation of the proximal tibia, all soft tissues and fibulae were removed. After assembling the extramedullary tibia alignment guide in correct rotation, the cutting block was positioned using the primary tibia stylus touching the less affected side of the tibia. The resection depth of the proximal tibia was 8 mm with a posterior slope of 3°. The osseous bed was pulse jet lavaged (InterPulse Jet Lavage, Stryker, Duisburg, Germany) and dried before cement application. Cooled bone cement was prepared using a vacuum mixing system. For Palacos^® ^R+G bone cement the EASYMIX vacuum system (Heraeus, Wehrheim, Germany) and for Refobacin^® ^Bone Cement R Optivac^® ^vacuum system (Biomet, Warsaw, USA) and a vacuum pump were used. The goal was to achieve the best cement quality and to avoid variations in consistency by standardizing the cementation process. The cement was applied to all components with an injection gun which was part of the vacuum mixing systems. Palacos^® ^R+G was applied at 21°C 2 minutes after preparation and Refobacin^® ^Bone Cement R at 21°C 4 minutes after preparation, according to the manufacturer's guidelines. For horizontal application, the cement was applied on the undersurface of the tibial tray with a coating thickness of approximately 4-6 mm. In the full application group, the cement was additionally placed into the stem channel and spread on the stem surface. The original tibial tray was then impacted into the prepared osseous bed using a mallet and an impactor handle. Finally, pressure on the tibial tray was maintained for 15 minutes. Afterwards, the distal third of the tibiae were osteotomized and discarded in a standardized manner. Last, test specimens were firmly mounted into a computer controlled universal testing machine (Typ 81806, EDC-100, Frank, Weinheim, Germany). The tibial tray was axially loaded with 2000 N (approximately three times the body weight of a 70 kg patient, the approximate peak load during normal walking [7]) for 10,000 cycles. All experiments were carried out without rotation or angular stress to the tibial component. During biomechanical examination, the test specimens were moistened with physiological saline solution every 10 minutes.

After mechanical testing, tibial components were explanted by one surgeon. Implants were disconnected with a chisel at the cement-implant interface on the anterior side and at the medial and lateral compartments; the remaining cement was removed with a Stille-Luer forceps and chisel. The time to complete explantation was recorded. To measure the bony defect, specimens were scanned on a multislice computed tomography (MSCT) scanner (Somatom Definition, Siemens, Forchheim, Germany); including the prosthesis and after extraction of the prosthesis. The following imaging parameters were used: 0.6 mm collimation, tube voltage 140 kV, tube current time product 300 mAs. Images were reconstructed with a slice thickness of 0.6 mm and a reconstruction increment of 0.4 mm, using a B70 s-Kernel and an extended Hounsfield-Scale (-10240-30710 HU). For digital image processing the clinical Workstation (MultiModality Workplace Version VE31A, Siemens, Forchheim, Germany) with the Volume software package was used. The dataset was loaded into the software, and thresholds for Hounsfield-Units (HU) were defined for bone (-500-2000 HU) and prosthesis (2000-30710 HU). Volumes for each bone enclosing the implant, as well as the remaining bone were calculated.

## Statistical analysis

For comparison of bone defects between cement types and techniques, a two-way ANOVA test without interaction was used. Tukey's Honest Significant Difference method was used to obtain 95% family-wise confidence intervals and adjusted p-values. A p-value of 0.05 or less was considered to be statistically significant. All data were analyzed using SPSS for Windows (version 11.0, SPSS Inc., Chicago, IL, USA).

## Results

**Figure 1:**
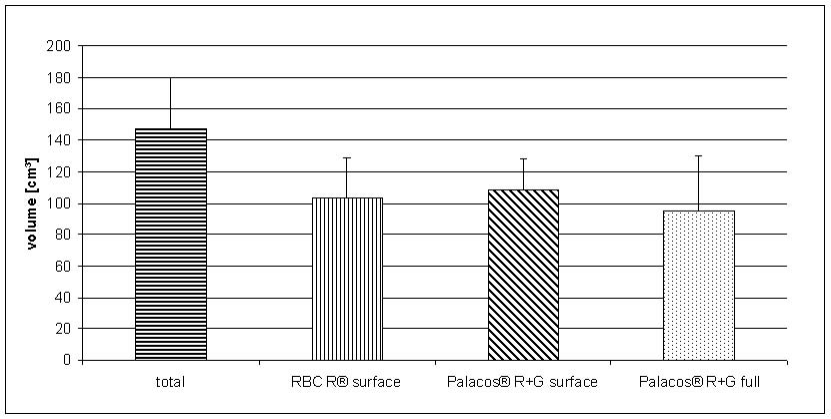
**Mean volume (cm^3^) of the remaining bone of the proximal tibia after removal of the prosthesis and bone cement in each of the three groups: surface cementing with Refobacin^® ^Bone Cement R, surface cementing with Palacos^® ^R+G, and full cementing with Palacos^® ^R+G**.

The CT measured mean volume of all specimens enclosing the prostheses was 147.2 ± 32.3 cm^3^. The volume of the remaining bone was 103.1 ± 25.7 cm^3 ^in the first group, 108.1 ± 19.8 cm^3 ^in the second group, and 95.1 ± 34.9 cm^3 ^in the third group. There were no significant differences in the degree of bone loss between cement techniques (p = 0.077; 95% CI -1.14 - 21.03) or cement types (p = 0.345; 95% CI -6.05 - 16.70) (Figure 1). The required time to explantation was 170.6 ± 54.89, 228.7 ± 84.5, and 145.7 ± 73.0 seconds in the first, second, and third groups, respectively.

## Discussion

We found no significant difference in tibial bone loss after explantation of the tibial component for either the two bone cement types, or cementing technique (full stem versus surface cementation of the tibial component).

Bone cement is commonly used for fixation of joint arthroplasties and transfers the acting strength of the implants on the bone. Bone cement must penetrate the cancellous bone in order to achieve micro-interlock and successful long-term prostheses survival. Nevertheless, cement is a weak interface [8,9] and has been shown to deform and degrade over time, resulting in debris, infection, and implant loosening [10]. Loosening of the tibial component is the major cause for failure in cemented TKA [11]. The interface between cement and bone was affected; most studies have focused on the evaluation of this interface [12-14]. In the literature, two areas have been investigated. While the component surface is one of the factors which place the cement-prosthesis interface at risk, technique, osseous bed preparation, and prearrangement and application of the acrylic cement were important for tibial component survival [11,15-18]. Sharkey et al. [19] reported a 10.5% loosening rate of the surface cemented components, and therefore impressed significantly higher than the fully cemented stem. Fully cemented stems were more difficult to remove during revision and were accompanied by increased bone loss in the metaphyseal area. Furthermore, the type of bone cement appeared to be important for long-term stability of the implant [6].

The use of polymethyl metacrylate (PMMA) bone cement worldwide has increased over the last decades [20]. Manufacturers produce cements of varying viscosities, mechanical, and physical properties [20,21]. Varying properties of bone cements can lead to different levels of penetration into the tibia apophysis. In vitro studies demonstrated a significant influence of penetration on the initial component stability in TKA [11,22,23]. Limited experimental and clinical data exist for the material properties of the Refobacin^® ^Bone Cement R. Kock et al. [6] compared the chemical composition, handling properties, and ISO standard mechanical testing of four different bone cements in vitro. Chemical analysis showed that the copolymers in Refobacin^® ^Bone Cement R differed from the Palacos^® ^cements. Furthermore, they noticed significant differences in their material properties, such as viscosity and waiting time for application; however, all cements were compliant with the required mechanical testing properties according to the ISO standard. Despite these reported differences in material properties, we found no significant difference due to bone loss during revision of the tibial component. This implies that previously reported differences in bone cements have no effect on cancellous bone penetration, and therefore no effect on the level of the bone defect in revision surgery secondary to the horizontal cementing technique.

One controversial aspect in TKA is the fixation technique of the tibial component. Full cementation including the tibial stem has good results and is currently the gold standard [10]. However, revision of a fully cemented stem is often accompanied by extensive tibial bone loss and therefore presents a more difficult scenario for reimplantation of a revision device. Due to these issues and the lack of scientific verification of the advantages for cementation of the tibial stem, surface cementing of the tibial tray is common.

Bert and McShane [22] evaluated surface and stem cementation on synthetic tibiae in vitro. They found improved implant stability by adding cement around the tibial stem, unless the cement mantle beneath the tibial component was increased up to 3 mm. However, using a 3 mm cement mantle under the tibia base plate may be clinically impractical [22]. On the contrary, further investigations evaluating the effect of surface cementation versus full tibial component fixation in cadaveric tibiae showed no difference between cementation techniques. Peters et al. [11] postulated that a cement mantle of 3.6-4.9 mm in all specimens was responsible for this finding. In contrast, Luring et al. [24] found a significantly increased maximum lift off when only the surface of the tibial baseplate was cemented, when comparing full-stem and surface cemented tibial trays. They concluded that cementation of the stem was necessary to prevent increased micromotion and early loosening.

The primary aim of our study was to investigate bone loss after revision of the tibial component implanted using the horizontal and full cementing techniques. CT could not demonstrate significant differences in bone loss between these two cementing techniques. These findings could be a result of shaft preparation of the tibial component, dispersion of applied bone cement, and potentially the direction of the applied implantation force causing cement intrusion. While intrusion of bone cement in the cancellous bone at the proximal tibia occurs primarily under the base plate of the tibial component, cement intrusion into cancellous bone along the tibial stem appeared to be less. For this reason, we investigated whether different bone cements could cause different levels of bone loss at this region of interest. However, bone loss did not correlate with different bone cements using the horizontal cementing technique. It can be assumed that cement intrusion into the bone along the tibial stem will be less and will not cause larger bone defects.

The limitations of this study are mainly in the experimental setup. First, the use of cadaver bone has a missing bone response. Second, the limited weight bearing capacity of 10,000 cycles only represents the clinical scenario 6 weeks postoperatively. These two factors reflect early loosening after TKA. This study, simulating bone defects in revision TKA and exploring the effects of different bone cements and cementation techniques, is the first of its kind. Factors influencing implant loosening, such as bone response, wear debris, biomechanical stress, infection, and inflammation were not considered and require further investigations.

## Conclusion

In this study, different cement techniques and bone cement types have no effect on bone loss in simulated revision surgery of the tibial component in TKA. Our findings should be verified by further investigations to identify the most effective and favorable cementation technique in TKA.

## Competing interests

The authors declare that they have no competing interests.

## Authors' contributions

TE participated in the study design, interpretation of results, and manuscript drafting. JF performed the CT scans and helped with analysis of radiological data. DS performed the study and participated in interpretation of the results. SFW participated in the study design and interpretation of the results. COT helped to draft the manuscript and interpret results. NT performed the statistical analysis. JRJP set up the protocol and participated in the study design. AS participated in the study design, performed the study, interpreted results, and helped to draft the manuscript. All authors read and approved the final manuscript.

## Pre-publication history

The pre-publication history for this paper can be accessed here:

http://www.biomedcentral.com/1471-2474/12/6/prepub
